# RNA Structural Elements of Hepatitis C Virus Controlling Viral RNA Translation and the Implications for Viral Pathogenesis

**DOI:** 10.3390/v4102233

**Published:** 2012-10-19

**Authors:** David Piñeiro, Encarnación Martinez-Salas

**Affiliations:** Centro de Biología Molecular Severo Ochoa, Nicolas Cabrera, 1, Cantoblanco, 28049 Madrid, Spain; Email: dpineiro@cbm.uam.es

**Keywords:** hepatitis C, translation control, IRES, RNA structural elements, host factors

## Abstract

Hepatitis C virus (HCV) genome multiplication requires the concerted action of the viral RNA, host factors and viral proteins. Recent studies have provided information about the requirement of specific viral RNA motifs that play an active role in the viral life cycle. RNA regulatory motifs controlling translation and replication of the viral RNA are mostly found at the 5' and 3' untranslated regions (UTRs). In particular, viral protein synthesis is under the control of the internal ribosome entry site (IRES) element, a complex RNA structure located at the 5'UTR that recruits the ribosomal subunits to the initiator codon. Accordingly, interfering with this RNA structural motif causes the abrogation of the viral cycle. In addition, RNA translation initiation is modulated by cellular factors, including miRNAs and RNA-binding proteins. Interestingly, a RNA structural motif located at the 3'end controls viral replication and establishes long-range RNA-RNA interactions with the 5'UTR, generating functional bridges between both ends on the viral genome. In this article, we review recent advances on virus-host interaction and translation control modulating viral gene expression in infected cells.

## 1. Introduction: The HCV Viral Genome

Hepatitis C virus (HCV) is a member of the Hepacivirus genus, within the *Flaviviridae* family, responsible for non-A non-B hepatitis, affecting about 3% of the human population worldwide. HCV causes persistent infection leading to severe liver damage, including cirrhosis, hepatic steatosis and hepatocellular carcinoma. The virus is predominantly transmitted through the parenteral route during contaminated blood transfusions as well as needle sharing among injecting drug users. Currently, there is no vaccine against HCV. Medical treatment of infected patients with pegylated interferon (IFN)-a and ribavirin is effective only in 40% of the infected population. More recently, a combination of this therapy with viral protease-inhibitors has improved virus clearance, reaching 60% of the HCV patients [1]. However, efficacy of the treatment depends on the particular HCV genotype [2]. It is well established that the HCV viral genome displays a large genetic variability [3,4,5,6]. This property is shared with other RNA viruses [7]. There are six different HCV genotypes, each one including multiple variants arising during viral replication as a consequence of the lack of proofreading activity of the viral RNA‑dependent RNA polymerase. Furthermore, the high mutation rate of this virus facilitates the emergence of resistant viral genomes during therapeutic treatment [8].

**Figure 1:**
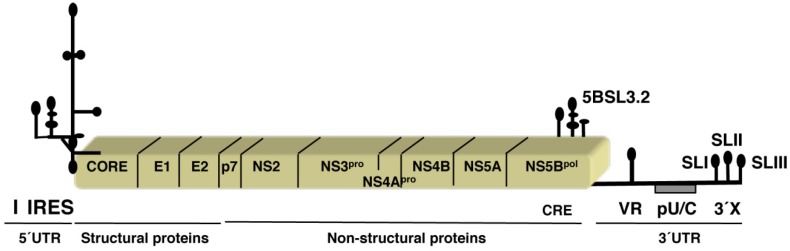
Diagram of the hepatitis C virus genome. Processing of the single polyprotein renders the mature viral proteins Core, E1, E2, p7, NS2, NS3, NS4A, NS4B, NS5A and NS5B. The stem-loop I at the 5' untranslated regions (UTR) and the CRE element located within the coding region of NS5B are required for viral replication. The structural elements of the 3'UTR region referred to in the text (the variable region VR, the polyU/C tract pU/C, and the 3'X region with stem-loops SLI, SLI, and SLIII) are schematically represented.

The viral genome consists of a single-stranded RNA of positive polarity, about 9.5 kb in length (Figure 1). Untranslated regions (UTR) located at the 5' and 3'end of the genome flank a single open reading frame (ORF), which encodes a polyprotein of about 3,000 amino acids [9]. The polyprotein is processed by viral and cellular proteases rendering the mature viral proteins. Structural proteins (core, E1, E2) are encoded in the most N-terminal region of the ORF, while the non-structural (NS) proteins (p7, NS2, NS3, NS4B, NS5A and NS5B) reside in the most C-terminal region. Core is a multifunctional protein that modulates viral and cellular gene expression, in addition to forming part of the viral nucleocapsid. E1 and E2 are the envelope glycoproteins that control receptor-mediated cell entry. P7 is an essential protein that appears to form an ion channel in lipid bilayers. NS2 is a cysteine protease while NS3 is a serine protease within its N-terminal and possess helicase and NTPase activity. NS3 together with NS4A are responsible for the processing of the viral polyprotein between NS3/4A, NS4A/B, NS4B/5A and NS5A/B. NS4B induces membranes in infected cells associated to the viral replication process; NS5A cooperates in viral replication with NS5B, the viral RNA-dependent polymerase. Additionally, NS5A has been implicated in the modulation of the IFN response.

In common with other single-stranded positive-sense RNA viruses, the 5' and 3'UTRs of HCV contain essential structural motifs that exert critical regulatory functions, controlling viral RNA translation and replication [10,11]. The viral RNA is uncapped; instead, the 5'UTR contains an RNA regulatory region termed internal the ribosome entry site (IRES) element (Figure 1), which drives translation initiation using a cap-independent mechanism. Additionally, the 3'end of the viral genome does not possess a poly(A) tail; rather, a poly(U/C) tract at the 3'end precedes a three stem-loops region termed 3'X [12]. Both 5' and 3'end regulatory elements perform crucial roles during viral multiplication that differ strongly from the mechanisms used by the host cell. Furthermore, viral protein synthesis is the first intracellular step of the virus multiplication cycle; thereby, these RNA motifs constitute candidate targets for antiviral molecules.

Most eukaryotic mRNAs initiate translation using a cap-dependent mechanism, by which the m^7^GpppN (cap) at the 5'end of the mRNA is recognized by the eukaryotic initiation factor (eIF)4F complex (consisting of the cap-binding factor eIF4E, the RNA helicase eIF4A, and the scaffold protein eIF4G that in turn, interacts with eIF4E, eIF4A, eIF3 and the poly(A)-binding protein (PABP)). This complex, bound to the 40S small ribosomal subunit, scans the 5'UTR of mRNA in 5' to 3' direction until an initiation codon is encountered in the appropriate context [13]. In contrast to this general mechanism used by host cell mRNAs, initiation of protein synthesis in various viral mRNAs (exemplified by picornaviruses) occurs internally, driven by IRES elements [14], using a mechanism that evades the inhibition of translation initiation usually occurring in infected cells. Early after the discovery of IRES elements in the genomic RNA of picornaviruses, an IRES element was reported to drive translation initiation of the HCV genomic RNA [15].

## 2. Structure and Function of the Hepatitis C IRES Element

Alignment of RNA sequences obtained from clinical isolates corresponding to the HCV IRES region indicated that, though variable, this is the most conserved region of the genome [16]. This feature that is commonly found in RNA regulatory motifs is indicative of the superposition of RNA structural constrains in this part of the genome. Accordingly, HCV IRES activity is strongly dependent on its RNA structure organization [17,18]. Evidence for tight links between RNA structure and biological function is provided by the conservation of structural motifs within IRES elements of highly variable RNA viruses [14,19]. Although some positive-strand RNA viruses (including Picornaviruses, Hepaciviruses and Pestiviruses) share the overall organization of their genomic RNA, IRES elements present in these viral genomes are organized in high-order structures that not only differ profoundly among them, but also with other viral IRES elements [20,21,22,23,24]. In all cases, RNA structure of viral IRES elements including HCV and the related GB virus B (GBV-B) is organized in modules that are phylogenetically conserved [25,26,27,28,29,30]. More specifically, in many RNA viruses it has been experimentally demonstrated that mutations leading to the disruption of specific RNA structure motifs impaired IRES activity while the corresponding compensatory mutations restored IRES function [31]. 

**Figure 2:**
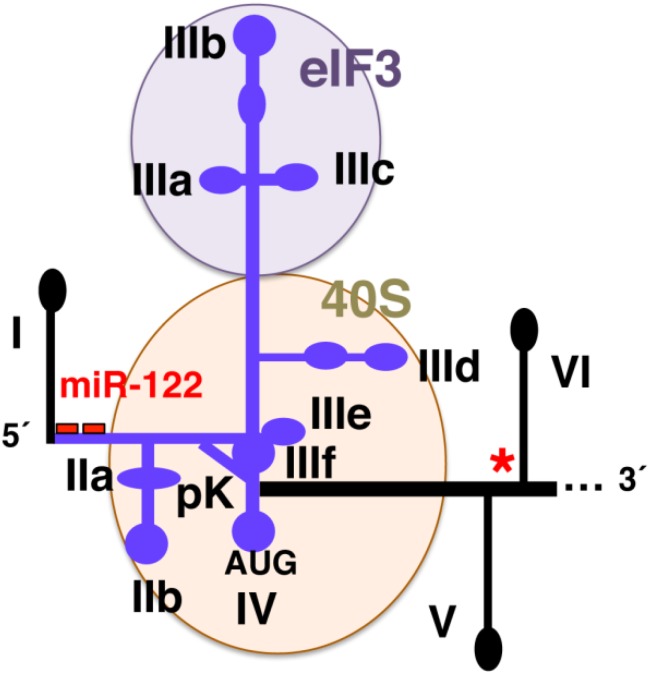
Schematic representation of the Hepatitis C virus (HCV) internal ribosome entry site (IRES) element and surrounding stem-loops. The IRES domains II, III, and IV, with stem-loops IIab and IIIabcdef, the pseudoknot (Pk), as well as the location of the initiator AUG codon in the loop of domain IV are depicted. The interaction sites of eIF3 and the 40S ribosomal subunit within the IRES element are highlighted. Domain I (involved in RNA replication) as well as the binding sites of miR‑122 are depicted by a hairpin and red bars, respectively. A red asterisk in stem-loop VI depicts a sequence potentially annealing with the target region of miR-122, proposed to lock the IRES element in a closed conformation.

The HCV IRES spans a region of approximately 340 nt that encompasses most of the 5'UTR of the viral mRNA and the first 30–40 nt of the core-coding region [31]. Variants of the 5'UTR isolated from HCV infected patients show distinct effects on IRES activity depending on their location within the RNA structure and the type of cell line used to determine IRES activity [26,27]. Specifically, the HCV IRES is arranged in conserved structural domains II, III and IV, from 5' to 3' direction, each of them organized in short subdomains (Figure 2). Besides these conserved stem-loops, a pseudoknot (pK) located upstream of the AUG start codon is a typical feature of the HCV IRES. This overall domain organization is conserved among Hepaciviruses, Pestiviruses [24,28] and some members of the *Picornaviridae* family that possess HCV-like IRES elements [22]. 

Given the critical role of the IRES element during HCV multiplication cycle, the RNA structural organization as well as the function of the distinct HCV IRES domains has been analyzed extensively. Domain II consists of two subdomains, IIa and IIb [32]. The RNA structure of subdomain IIa adopts an L-shape conformation essential for IRES activity [33]. This subdomain appears to direct the apical loop IIb towards the E site of the ribosome (tRNA-exit site). Accordingly, it has been recently found that domain IIb affects the RNA conformation in the decoding groove of the 40S ribosomal subunit [34]. 

Domain III is the larger domain of HCV IRES (Figure 2); it consists of stem-loops IIIabcdef, organized in three and four-way junctions [35,36], which appear to have a division of functions. The entire domain III is required for positioning the mRNA start codon correctly on the 40S ribosomal subunit during translation initiation, but the IIIdef region is sufficient to form a high-affinity interaction binary complex with the 40S ribosomal subunit in the absence of eIFs. The apical region of this domain (IIIabc) contains the eIF3-binding site [37]. The basal region is organized in three stem‑loops (IIIdef) that can fold as a pseudoknot structure [38]. A particular feature of domain IIId conserved among all genotypes of HCV is the presence of an internal loop E motif [36], an RNA motif also present in domain II and other structured RNAs such as the 5S RNA. The three-dimensional structure of domain III solved recently reveals a complex pseudoknot that establishes the alignment of two helical elements on either side of the four-helix junction [39], constraining the orientation of the open reading frame for positioning on the 40S ribosomal subunit. 

From the mechanistic point of view, binding of the HCV IRES to the 40S ribosomal subunit is the first step of translation initiation on the viral RNA [40,41,42]. As mentioned above, interaction with the 40S subunit requires hairpins IIIcdef of the IRES [37], while domain II helps to accommodate the mRNA in the tRNA-exit site of the ribosome and mediates eIF2 release during 80S ribosome assembly [43,44]. Following assembly of the binary 40S-IRES complex, eIF3 and the ternary complex eIF2‑GTP-tRNA_i_ are recruited to form the 48S initiation complex [17]. In full agreement with these data, direct interaction of the polypeptides conforming eIF3 with the HCV IRES has been identified by different approaches, including UV-crosslink, mass spectrometry of IRES-bound protein complexes and cryo-electron microscopy [45,46,47,48,49]; furthermore, the ribosomal proteins that participate in IRES‑40S interaction have been identified by cross-linking and mass spectrometry [41,50]. 

This specific mode of internal initiation of translation is not unique to HCV; HCV-like IRES elements with similar eIF requirements have been found in the genome of some picornaviruses [51,52] as well as in the pestiviruses [18]. Specifically, the interaction sites with the 40S ribosomal subunit and eIF3 are conserved between HCV and HCV-like IRES elements. Furthermore, covariation between a purine-purine mismatch near the pseudoknot and the loop sequence of domain IIIe maintains a tertiary interaction that stabilizes the pseudoknot structure and correlates with translational efficiency in the HCV-like porcine teschovirus-1 talfan and HCV IRES elements [52].

Besides this structural and mechanistic conservation between pestiviruses and the HCV-like IRES of picornaviruses, there are features conserved with more distant IRES elements. Indeed, cryo-electron microscopy studies of the IRES elements of HCV and the intergenic region (IGR) of dicistroviruses (a pseudoknotted IRES element that does not need eIFs to recruit the 40S ribosomal subunit) showed that these RNAs are accommodated in the interface of the ribosomal subunits in a similar manner [44,53]. Furthermore, and despite the different structural organization of the IGR IRES of dicistroviruses and the HCV IRES elements, both interact with the ribosomal protein RpS25 [54] and induce similar conformational changes in the 40S ribosomal subunit. This characteristic opens the possibility that IRES elements could possess a structural motif mediating their direct interaction with the 40S subunit. 

The IRES property of interacting with the ribosome is an attractive possibility to explain internal initiation of translation, which is supported by the finding that IRES activity is differentially sensitive to changes in ribosome composition [55]. Another feature shared among the IRES elements of dicistroviruses, picornaviruses and pestiviruses is their recognition as substrate of RNase P, the tRNA precursor-processing ribozyme [56,57,58], a result that led to propose the existence of tRNA-like motifs within the HCV IRES element [59]. tRNA-like motifs, often present in RNA viruses, could be inherited from RNA replication signals accommodated to assist in the translation process. Indeed, plant RNA viruses make use of tRNA-like structures to control cap-independent translation initiation and viral RNA replication [60,61] assisted by RNA-RNA interactions between the 3' and the 5'UTR of the viral genome.

## 3. Host Factors Controlling HCV Protein Synthesis

Besides ribosomal proteins and eIFs, RNA-binding proteins that play important roles in gene expression help to promote IRES-dependent translation acting as IRES-transacting factors (ITAFs) [62]. Indeed, a number of riboproteomic approaches have facilitated the identification of proteins interacting with different IRES elements including HCV [48,49,63]. One of the proteins specifically identified in HCV IRES-40S ribosomal complexes is the receptor for activated protein kinase C (RACK1) [49], a protein that recruits protein kinase C to the 40S subunit. Despite the large differences in RNA structure organization, various ITAFs are shared between the IRES elements of HCV and picornaviruses, providing evidences of common characteristics between distant IRES elements that could be related to similar mechanisms of action. Amongst others, examples of common factors between HCV and picornavirus IRES elements are PTB, PCBP2, nucleolin, Gemin5, unr, hnRNPA1/A2, La autoantigen (La) and NS1-associated protein (NSAP1) [64,65,66,67,68]. In most cases ITAFs stimulate translation initiation (PTB), although there are examples of translation repressors (Gemin5) [67]. Whether these factors exert their function directly or through the action of other partners in the complex remains elusive. However, the fact that most of these proteins also contribute to other processes of the cellular gene expression program establishes a network of interactions linking translation control with other RNA-based events such as splicing, transport, or stability of cellular mRNAs. Most of these steps are altered in infected cells due to changes in localization, post‑translational modification or proteolysis of key components of the cellular machinery responsible for these processes [69].

In addition to eIFs, ribosomal subunits and RNA-binding proteins, HCV subverts the liver-specific microRNA miR-122 to upregulate viral RNA abundance [70]. miR-122 interacts with the 5' end of the HCV RNA genome (Figure 2) using two binding sites located 8 or 9-nucleotide apart, depending on the HCV isolate. Both binding sites are occupied by miR-122 and function cooperatively to regulate viral gene expression [71], forming a tandem oriented oligomeric RNA complexes assembled at the 5' terminus of the HCV RNA. In this complex, each miR-122 molecule utilize similar internal nucleotides to interact with the viral genome, creating a bulge and tail in the miRNAs [72], likely protecting the 5' terminal viral sequences from nucleolytic degradation or from inducing innate immune responses to the RNA terminus.

miR-122 stimulates HCV IRES-mediated translation [73]. In agreement with this, other authors proposed that the HCV IRES resides within a locked conformation in the naked RNA owing to the annealing of its flanking sequences (Figure 2), and that miR-122 unlocks this high-order RNA structure exposing the IRES element to an open conformation [74]. However, comparison of the replication capacity of a double miR-122 binding-site mutant and a defective IRES mutant suggests that the decrease in translation efficiency associated with loss of miR-122 binding does not account for the defect in virus production by the double mutant [75]. This result suggests that miR-122 could be acting at an additional step in the virus life cycle. In this regard, it has been recently reported that miR‑122 positively regulates the viral life cycle in culture cells by stimulating HCV translation mediated by Argonaute proteins through a process that requires uncapped RNA, the HCV IRES and the 3' region of miR-122 [76]. This mechanism of translation activation by an Ago-containing miRISC complex differs from the one described earlier [74], proposed to occur *in vitro* using naked RNA.

## 4. Interference with HCV Translation Inhibits Viral Multiplication

The large difference in the mechanism used by the HCV RNA to initiate translation makes this step of the viral multiplication cycle an ideal target in the development of antivirals. With this aim, many investigators have explored the inactivation of the virus by the development of therapeutic approaches involving direct targeting of the IRES region as well as the interference of specific host factors required for viral RNA translation. Attempts to block IRES activity directly with antisense oligonucleotides, peptide nucleic acids (PNAs), locked nucleic acid (LNAs), DNA and RNA aptamers, RNAi and ribozymes have been widely explored [77,78,79,80]. Of interest, an LNA antisense oligonucleotide with potent antiviral activity targeted the region containing both miR-122 binding sites [81], supporting the critical role of this part of the genome in controlling the viral multiplication cycle.

Screening of small molecule inhibitor libraries has yielded limited success to control HCV multiplication [82]. However, a benzimidazole molecule that binds to HCV IRES subdomain IIa [83] reduces viral protein expression and inhibits viral RNA replication [84]. Structural studies aimed at understanding the mechanism of action of this small molecule indicated a conformational change of domain II induced by the binding of the drug that impaired IRES activity [85,86]. The recent resolution of the three-dimensional structure of the IRES subdomain IIa in complex with this molecule revealed a binding mode that resembles the substrate binding sites in riboswitches [87], explaining its interference with the translation process.

Although many attempts to inhibit different steps of the HCV multiplication cycle have been done *in vitro*, using cell-free systems or tissue culture cells, the lack of a convenient laboratory animal model has hampered the development of effective therapies. Interestingly, expression of two human genes, CD81 and occludin, has been shown to be sufficient to allow HCV infection of fully immunocompetent inbred mice [88]. This animal model opens new avenues for studying viral pathogenesis in addition to provide a platform for testing HCV therapeutic drugs. 

## 5. Implications of Viral RNA 5'-3' End Interactions on IRES Activity and the Infection Cycle

The untranslated regions of the genome of positive-strand RNA viruses contain phylogenetically conserved functional elements that play essential roles during the viral cycle. These specialized structural motifs are recognized by viral and host RNA-binding proteins that control translation, replication and interaction of viral replication complexes with host cell structures. These processes have been studied in great detail in HCV that harbors RNA structural elements on each end of the viral genome (Figure 1). Interestingly, functional links between these distant regulatory regions has been shown to affect HCV gene expression. Thus, besides the regulatory function of the IRES region in translation initiation of the viral RNA, a translation enhancer function of the HCV 3' UTR variable region, the poly(U/C) tract, and the 3' terminal stem-loop 1 of the conserved 3'X region (Figure 1) was reported [89]. Along this idea, long-range RNA-RNA interactions between the 5' and the 3'end of the viral genome were observed [90]. This interaction specifically involved the apical loop of domain IIId of the IRES (Figure 2) and the internal loop of 5BSL3.2 within the *cis*-acting replication element (CRE) (Figure 1), a structural region that is essential for the initiation of viral replication [91]. The 5BSL3.2 motif forms part of a cruciform structure at the 3' end of the NS5B coding sequence that contributes to the three-dimensional folding of the 3' end of the genome by generating a kissing loop with the SL2 of the 3'X region [92]. 

A functional role of the IRES-5BSL3.2 long-range RNA interaction in translation control has been recently documented [93]. Mutations in 5BSL3.2 motif lead to an increase in IRES activity, supporting the existence of a high order structure in the HCV genome that involves domain IIId in the IRES and stem-loop 5BSL3.2. Other authors, however, have not detected this long-distant interaction [94]. The tertiary structure of the 3'UTR of HCV is not known yet. It appears that different long-range interactions may exist in this region, with significant differences between distinct HCV genotypes, as revealed by RNA structure mapping of this region by selective 2'-hydroxyl acylation and analyzed by primer extension (SHAPE) methodology [94]. This study has proposed a regulatory function of the 5BSL3.2 RNA motif in the switch from translation to replication of the viral RNA, two mutually incompatible events that use the same template.

Genome circularization promoted by direct RNA-RNA contacts involving inverted complementary sequences has been shown to be essential for viral replication in the case of flaviviruses [95]. In other RNA viruses, RNA-RNA interaction between the 3' and the 5' UTR sequences of the genome control cap-independent translation initiation [96]. Similarly, a functional link between the IRES and the 3' end of the viral RNA was earlier suggested by the specific stimulation of the aphthovirus IRES activity by the viral 3' UTR [97]. This link could take place through direct RNA-RNA contacts, through protein bridges mediating RNA circularization, or both [98,99]. Notwithstanding, host RNA-binding proteins NF90/NFAR mediate 5'-3' contacts in the genome of HCV and pestiviruses [100,101]. 

In the search of factors that could generate functional bridges between the ends of the HCV viral genome, it was found that the insulin-like growth factor II mRNA-binding protein 1 (IGF2BP1) is present in complexes assembled with RNAs that contained both the HCV IRES and the 3'UTR. This protein coimmunoprecipitates with eIF3 and the 40S ribosomal subunit [63] suggesting that it enhances HCV IRES activity by recruiting the ribosomal subunits to a pseudo-circularized RNA. These results provide a mechanistic basis for translation stimulation and replication of the viral RNA resembling the synergistic stimulation of cap-dependent translation. Furthermore, expression of DDX6 (Rck/p54, a member of the DEAD-box RNA helicase family) is required for optimal HCV replication. Overexpression of DDX6 enhanced replication, whereas a helicase-deficient mutant had a dominant-negative effect reducing HCV yields. Formation of a complex containing DDX6, HCV core protein, and both viral and cellular RNAs, was dependent upon the C-terminal domain of DDX6 [102]. In this respect, requirement of HCV RNA translation and replication on Rck/p54, LSm1, and PatL1, which regulate the mRNA decay pathway, was linked to the 5' and 3' UTRs of the viral genome [103]. 

## 6. Modulation of Viral and Cellular Gene Expression in Infected Cells: Implications for Viral Pathogenesis

HCV, as all RNA viruses, depends on the host cell machinery to synthesize the proteins necessary for viral replication. Consequently, RNA viruses have developed specialized strategies to subvert the cellular machinery in their own benefit, including cellular translation factors and signaling pathways that control host protein synthesis [69]. In turn, the cell has evolved responses to recognize and fight the incoming infectious agent. Cells infected by RNA viruses accumulate double-stranded RNA (dsRNA), leading to the activation of the cellular RNA-dependent protein kinase (PKR), a protein that has a key role in the innate immunity response to viral infection [104]. PKR is responsible for phosphorylation of eIF2a, causing a reduction of eIF2.GTP.Met-tRNA_i_ ternary complex, and thus inhibiting mRNA translation [13]. To counteract this response, RNA viruses have evolved multiple strategies. In addition of using a cap-independent IRES-dependent translation initiation mechanism, HCV mRNA evades the inhibitory effects of eIF2α phosphorylation by using an alternative initiator tRNA-binding protein, eIF2A. HCV RNA recruits eIF2A to the HCV IRES via direct interaction with the IIId domain and subsequent loading of Met-tRNA_i_ to the P site of the 40S ribosomal subunit [105]. Furthermore, HCV RNA translation is independent of eIF2 under high concentrations of MgCl_2_ in cell free systems [106]; whether this also occurs in infected cells is unknown. 

An alternative way used by RNA viruses to control the translation repression effect induced by the host stress response to infection is to take advantage of the oscillation in this response to prevent continuing translation repression [107]. Virus infection-induced translation inhibition is linked to assembly of stress granules (SGs). HCV infection induces a dynamic assembly of SGs, concomitant with phases of active and stalled translation, delayed cell division, and prolonged cell survival. eIF2α phosphorylation by PKR mediates SG formation and translation arrest. This event is antagonized by the upregulation of GADD34, the regulatory subunit of protein phosphatase 1 dephosphorylating eIF2α. At least in part, these oscillations are reminiscent of the fluctuations in cell viability and virulence observed during coevolution of cells and viruses in other persistent infections, such as foot‑and-mouth disease virus [108]. The latter establishes persistent infections in carrier animals and in tissue culture cells and uses IRES-dependent translation to drive viral protein synthesis while inactivating host cell gene expression by proteolysis of translation factors and RNA-binding proteins [99,108,109,110]. However, HCV infection does not trigger the proteolysis of eIFs and RNA-binding proteins needed for cellular mRNA translation [111]. These observations suggest that viral infection can establish persistent infection by using different manners to coordinate host cell proliferation and viral gene expression.

HCV-induced persistent infection reflects the evasion of host immunity and interference with IFN innate immune defenses. Cellular antiviral immunity, among others, is based on the host recognition of virus dsRNA intermediates, which are sensed by Toll-like receptor 3 (TLR3) and the RNA helicase RIG-I (retinoic acid inducible gene I). Detection of dsRNA results in the activation of the transcription factors interferon regulatory factor 3 (IRF3) and NF-kappaB. CARD-adaptor proteins interact with RIG-I and recruits IKK kinases, leading to the activation of NF-kappaB and IRF3. However, the HCV NS3-4A viral serine protease cleaves cardif (a CARD-adaptor protein also known as the mitochondria-antiviral signaling protein MAVS) [112] as well as the Toll-IL-1 receptor domain-containing adaptor inducing IFN-beta (TRIF) [113] interfering with host innate immune defenses by blocking IFN-b production. Consistent with this, PKR inhibition restored IFN induction in infected cells until a decrease in IFN expression could be attributed to NS3/4A-mediated MAVS cleavage [114]. 

The response of the cell to HCV infection affects various pathways that may differ between individual patients, determining the outcome of the infection. In fact, both cytopathic effects and immune-mediated processes play an important role in liver injury. At least in part this has been illustrated by cell lines that have different permissiveness to infection and show a differential expression of genes linked to the innate immune response, secreted signal peptides and host factors that have a role in virus entry and replication [115]. On the other hand, the induction of cell-death related genes and the activation of caspase-3 in HCV-infected cells is coincident with viral replication, suggesting a link between viral load and apoptosis [116]. Furthermore, transgenic mice expressing HCV proteins recapitulate the hallmarks of HCV infection [117]. Consistent with this, interactions of HCV-encoded proteins with cell cycle regulators and tumor suppressor proteins suggest that HCV can alter cellular proliferation control [118]. Further studies aimed at understanding the interplay between virus replication and cell response to infection are needed to shed light on this important persistent human pathogen.

## 7. Perspectives

Understanding the biological role of RNA regulatory elements present in the HCV genome has received great attention in the last years. In particular, the concerted action of these signals alongside host and viral factors is essential to control the viral multiplication cycle and at the same time to maintain the permissiveness of the cell to a sustained persistent infection. Mechanistic aspects that explain at the molecular level how HCV RNA structures and viral proteins participate in the infection process still need to be investigated in detail. In particular, efforts towards identifying functionally relevant interactors of the viral RNA regulatory elements, as well as viral proteins, will provide information about their mechanism of action. An important question is how these functional structures control the switch from translation to replication and later to encapsidation of the viral genome. In recent years, it has become evident that dynamic conformational changes controlled by RNA-RNA, RNA-protein and miRNA-RNA interactions modulate viral replication in the viral genome. This view of the viral genome as a flexible molecule will help in understanding the function of RNA structures formed at different stages of the viral life cycle. Deciphering how these high order structures are assembled in the context of the whole viral genome and how these macromolecular complexes govern the viral multiplication cycle is ahead of us.
